# PDGF-BB as a potential biomarker for early diagnosis of postpartum hemorrhage

**DOI:** 10.3389/fgwh.2026.1849806

**Published:** 2026-06-17

**Authors:** Karthikeyan Thirugnanam, Brock E. Polnaszek, Kevin Eng, Amy Y. Pan, Ramani Ramchandran

**Affiliations:** 1 Division of Neonatology, Department of Pediatrics, Developmental Vascular Biology Program, Medical College of Wisconsin, Children’s Research Institute (CRI), Milwaukee, WI, United States; 2 Division of Maternal Fetal Medicine, Department of Obstetrics and Gynecology, Medical College of Wisconsin, Milwaukee, WI, United States; 3 CIAN, Inc., Pewaukee, WI, United States; 4 Division of Bioinformatics and Quantitative Child Health, Department of Pediatrics, Medical College of Wisconsin, CRI, Milwaukee, WI, United States

**Keywords:** biomarker, bleeding, cilia, endothelial, placenta

## Abstract

Postpartum hemorrhage (PPH) is a leading cause of maternal morbidity and mortality, highlighting the need for effective methods to detect early identification and intervention. Previous data demonstrates that endothelial cilia, a microtubule-based organelle is responsible for vascular stability. Thus, we rationalized that proteins inside endothelial cilia may be candidates for conditions affected by dysregulated flow and barrier formation. Protein expression of four ciliary-associated candidates, including Platelet-Derived Growth Factor-BB (PDGF-BB), were quantified using Western blot and enzyme-linked immunosorbent assay (ELISA). Baseline characteristics were compared across groups, and logistic regression and receiver operating characteristic (ROC) analyses were performed to evaluate the association between PDGF-BB levels and PPH risk. Baseline characteristics were comparable across groups, except for maternal race and pre-pregnancy body mass index. Among the proteins evaluated, PDGF-BB demonstrated consistent differential expression across both analytical platforms. Higher PDGF-BB levels were independently associated with a lower risk of PPH in both unadjusted and adjusted models (adjusted OR 0.62, 95% CI 0.44–0.88; *p* = 0.0068). ROC analysis identified a PDGF-BB threshold of 11.5 pg/mL, yielding high discriminatory performance for PPH. These findings may suggest that low levels of PDGF-BB may be associated with risk of PPH, providing a potential early biomarker for this condition to inform management. Further prospective studies are needed to confirm the temporal relationship, causal pathway, and diagnostic utility of PDGF-BB for risk of PPH.

## Introduction

Postpartum hemorrhage (PPH) remains a leading cause of maternal morbidity and mortality worldwide, with substantial implications for women's health outcomes (1). According to the World Health Organization (WHO), each year, approximately 14 million women experience PPH resulting in about 70,000 maternal deaths globally (2). Defined as a blood loss of ≥ 1000 mL following childbirth, PPH can occur suddenly and with little warning, complicating approximately 1%–3% of all deliveries worldwide (3). Recently, WHO has proposed lower diagnostic thresholds of > 300 mL blood loss for PPH diagnosis (4), which means that the numbers of PPH based on this definition is likely to increase. Despite advances in obstetric care, early prediction of PPH remains a major challenge, and is largely dependent on clinical assessment, which may be subjective and delayed (5). Existing risk stratifying tools demonstrate limited accuracy identifying 60%–85% of patients who will experience a PPH, while misclassifying 40% of patients who will not have a PPH (false-positive) and 1% of patients who do have a PPH (false-negative) (6). There remains a need for reliable biomarkers to accurately predict risk for PPH. Reliable biomarkers that can accurately predict risk of PPH may allow for early, timely management of PPH, ultimately improving maternal outcomes.

PPH is a failure of one of three vascular control systems, namely, myometrial mechanical compression, decidual/spiral artery thrombosis and placental bed vascular remodeling (7). Thus, the uterine-placental vascular system that includes the endometrium, myometrium and placental vasculature must work in concert to prevent PPH. Endometrium is a critical tissue involved in PPH as the endometrial vasculature acts within the uterine-placental vascular context (8). Endometrium also contains the spiral arteries and placental implantation vasculature, that must be rapidly closed post delivery (8). Thus, collectively, vascular remodeling is a key feature during pregnancy and delivery that requires precise control and homeostasis mechanisms, which when dysregulated can result in placental pathologies (preeclampsia (PE), placental disruption) that increase risk of PPH or can directly influence remodeling of spiral arteries to affect function (ineffective collapse upon myometrium contraction), leading to PPH.

Our laboratory extensively investigates the role of endothelial cell-derived molecules that participate in endothelial barrier function, with those associated with cilia, a microtubule-based organelle (9–11). Endothelial cilia participate in both flow-mediated mechanisms (12–16) and in vascular barrier formation (9, 11, 17–19). Endothelial cilia also control mural cell recruitment to promote vascular stability (10, 20). Finally, we showed that under compromised flow conditions, deciliation or physical removal of cilia occurs in blood vessels, and thus ciliary proteins may serve a biomarkers of altered flow or vascular injury (21). We therefore hypothesized that ciliary proteins involved in vascular stability (and flow mechanisms) may be found in blood of patients who have experienced PE, a risk factor for PPH or in PPH wherein vascular stability is compromised. We investigated this hypothesis in retrospective samples from pregnant women collected at the Medical College of Wisconsin's Maternal Placenta & Cord (MPC) Bank Milwaukee, Wisconsin, USA.

## Materials and methods

### Ethical considerations

This study was approved by the Institutional Review Board (IRB) of the Medical College of Wisconsin (PRO46125) under title, “Cilia protein detection in plasma samples”.

### MPC Bank

The MPC Bank housed 12,680 samples in 2022 over the time frame for samples used in this study. Participants in the MPC Bank are pregnant individuals aged ≥ 18 years who can read and speak English and provide informed consent. Enrollment includes a one-time maternal blood collection during routine prenatal care and permission to store any residual blood or placental tissues otherwise discarded after delivery, resulting in a large, heterogeneous biobank. Blood samples were collected via venipuncture into ethylenediaminetetraacetic acid (EDTA) tubes.

### Study design, participants and criteria for selection

We conducted a retrospective case–control study using biospecimens from the MCW MPC Bank. A total of 80 participants were selected as a pilot cohort and stratified into four groups based on delivery outcomes: PPH (*n* = 23), PE (*n* = 41), PE + PPH (*n* = 6), and controls without PE or PPH (*n* = 10). Maternal blood samples were collected antepartum at a median gestational age of 27 weeks 5 days (interquartile range 1 week 5 days). PPH was based on ACOG definition of > 1000 mL and quantitative blood loss using validated weighted protocols as recommended by the California Maternal Quality Care Collaborative (CMQCC). Inclusion criteria included availability of plasma samples and documented clinical outcomes at delivery. The study design was hypothesis-driven, focusing on conditions associated with altered vascular flow, including PE.

### Post tissue bank sample processing

Samples were processed immediately by centrifuging at 1500 x g for 10 min at 4 °C to separate plasma, which was stored at −80 °C until analysis. All sample collection procedures were conducted in accordance with institutional guidelines and ethical standards.

### Western blot analysis

Western blot analysis was performed to assess qualitatively the relative protein expression of four ciliary-associated proteins ADP-ribosylation factor-like protein 13b (ARL13b), gamma-tubulin (*γ*-tubulin), intraflagellar transport protein-88 (IFT-88) and PDGF-BB (10, 22). Three samples from each cohort for western was randomly picked and was the initial analysis performed before ELISA. Plasma samples (20 µL) were subjected to protein quantification using the BioRad DC protein assay followed by detection in a SpectraMax 340PC absorbance microplate reader. The plasma samples were diluted with RIPA buffer (Sigma) with complete mini EDTA-free protease inhibitor cocktail (Roche, Basel, Switzerland) and PhosSTOP phosphatase inhibitor (Roche) using a Qiagen TissueRuptor (Hilden, Germany). 20 μg of protein was loaded in sodium dodecyl sulfate-polyacrylamide gel electrophoresis (SDS-PAGE) gradient gel. The separated proteins were transferred to a polyvinylidene fluoride (PVDF) membrane, which was blocked with 5% non-fat dry milk in Tris-buffered saline containing 0.1% Tween 20 (TBST). Membranes were incubated overnight at 4 °C with a primary antibody specific to PDGF-BB (1:500, Novus Biologicals, Cat#NBP1-58279), ARL13B (1:500, Proteintech, Cat#17711-1-AP), IFT-88 (1:1000, Thermofisher, Cat#PA5-18467), *γ*-tubulin (1:1000, Genetex, Cat#GTX113286) and *β*-actin (1:1000, Cell signaling technology, Cat# 8457) followed by incubation with anti-rabbit horseradish peroxidase (HRP)-conjugated secondary antibody (1:1000, Cell signaling technology, Cat# 7074)and anti-goat HRP-conjugated secondary antibody (1:2000, Jackson Laboratory, Cat#205-052-176). The bands were visualized using enhanced chemiluminescence (ECL) detection, and band intensity was quantified using ImageJ software.

### Enzyme-linked immunosorbent assay (ELISA)

Quantitative analysis of PDGF-BB protein levels was performed using a commercially available research use only (RUO) ELISA kit (RayBiotech, Norcross, GA, Cat# ELH-PDGFBB-1). Plasma samples were thawed and diluted according to the manufacturer's instructions. A standard curve was generated using known concentrations of recombinant PDGF-BB, and sample concentrations were calculated by comparing their absorbance values at 450 nm to the standard curve according to an Excel file template provided by the company. All samples were measured in duplicate to ensure accuracy, and the inter-assay coefficient of variation (CV) was < 10%.

### Statistical analysis

Descriptive statistics were used to summarize the baseline characteristics of the study groups. All data are presented as *n* (%) or mean ± standard deviation (SD) or median and interquartile range. Shapiro–Wilk test was used to test normality and Levene's test was used to check the homogeneity of variance assumption. The normality test was performed within each group to ensure each group meets the assumption of normal distribution. Although the total cohort is 80, subgroup sample sizes (6–41) are small. Shapiro–Wilk test offers higher power and is more reliable for smaller sample size. Demographics were compared among the groups by analysis of variance (ANOVA), Kruskal–Wallis's test or Fisher's exact as appropriate. For group comparisons of PDGF-BB levels from western blot or ELISA, we used a one-way ANOVA or Kruskal–Wallis's test followed by Dunnett test or Dunn's test to adjust for multiple comparisons. Similar comparisons were made for other ciliary proteins in western blot. Logistic regression was performed to examine the association between PDGF-BB levels and development of PPH in PPH patients and controls. Pre-pregnancy BMI was included in the adjusted model. Odds ratio (OR) and adjusted odds ratios (aOR) with corresponding 95% confidence intervals (CIs) were calculated. Firth's penalized likelihood approach was used to address issues of separability. A *p*-value of < 0.05 was considered statistically significant. Statistical analysis was performed using SAS version 9.4 (SAS Institute Inc., Cary, NC) or SPSS version 29.0 (IBM Corp., Armonk, NY).

## Results

Baseline characteristics were generally comparable across groups, except for maternal race and pre-pregnancy body mass index. Pregnant patients who experienced a PPH, PE, or PPE + PE were more likely to be obese or self-identify as black compared to the control group (Table 1). The remaining baseline characteristics were not statistically different and include maternal age at delivery, gestational age at week of sample collection, or with other pregnancy comorbidities such as diabetes (Table 1). The sample groups did not have any known or unknown conditions thought to alter ciliary-associated protein levels in pregnancy.

**Table 1:** Demographic characteristics of study subjects.

Characteristics	Control (*n* = 10)	PPH (*n* = 23)	PE (*n* = 41)	PE + PPH (*n* = 6)	*P* value
Maternal age at delivery (years)	29.3 ± 5.4	29.5 ± 5.0	31.1 ± 5.6	32.8 ± 6.9	0.44
Maternal Race					0.043
Asian	0	0	1 (2.4)	0	
Black or African American	1 (10)	2 (8.7)	4 (9.8)	4 (66.7)	
White	9 (90)	21 (91.3)	35 (85.4)	2 (33.3)	
American Indian or Alaska Native	0	0	1 (2.4)	0	
Hispanic or Latino	0	0	1 (2.4)	0	0.43
Gestational age of sample (week)	27.4 (26.0, 28.1)	26.7 (25.7, 28.4)	27.7 (25.9, 31.0)	29.4 (26.3, 36.0)	0.42
BMI (pre-pregnancy)	24.4 (21.4, 27.8)	25.9 (22.4, 35.1)	30.4 (23.9, 36.3)	36.9 (29.9, 43.2)	0.028
Gestational diabetes	0	3 (13)	5 (12.2)	1 (16.7)	0.69
Gestational hypertension	0	4 (17.4)	13 (31.7)	1 (16.7)	0.14
Anemia	0	0	0	1 (16.7)	0.075
Placental previa	0	2 (8.7)	0	0	0.31
Polyhydraminos	0	1 (4.4)	1 (2.4)	0	> 0.99
Placental abruption	0	1 (4.4)	0	0	0.49
Macrosomia	0	0	0	1 (16.7)	0.075

Data are represented as mean ± standard deviation or median (interquartile range) and number (percentage).

### Western blot analysis shows increase in ciliary proteins levels and decrease in PDGF-BB levels compared to controls

**Figure 1:**
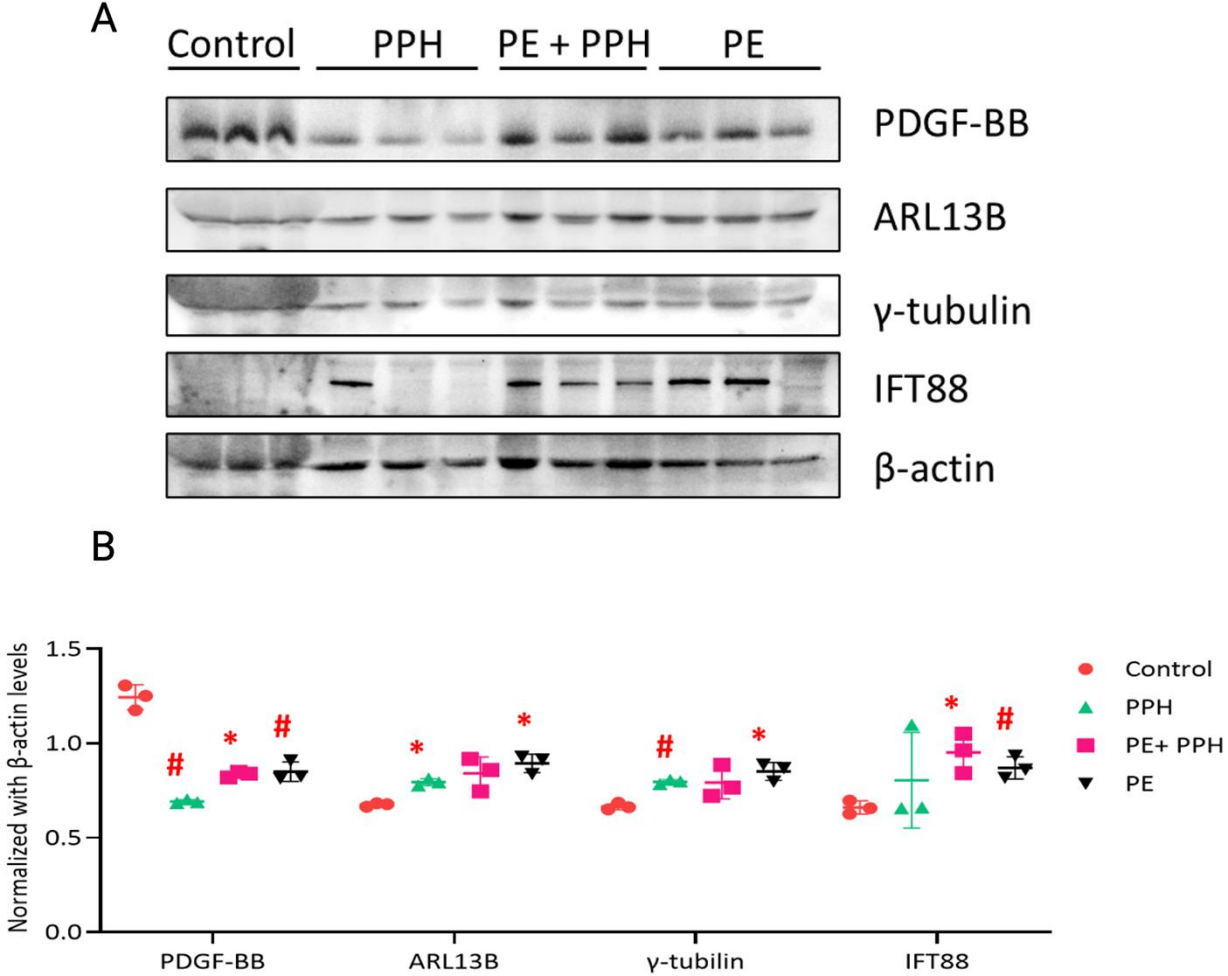
Western blot analysis of ciliary protein levels in human plasma samples. **(A)** Western blots for PDGF-BB, ARL13b, *γ*-tubulin, IFT-88 and *β*-actin proteins on control, PPH, PPH + PE, and PE human plasma samples are shown. Band sizes indicate proteins at 27 kDa (PDGF-BB), 48 kDa (ARL13b), 51 kDa (*γ*-tubulin), 120 kDa (IFT-88) and 45 kDa (*β*-actin). Blot probed for *β*-actin antibody were stripped and re-probed for all protein targets. **(B)** Western blots were quantified using ImageJ program, and normalized protein to *β*-actin levels are shown. *P < 0.05, #*P* < 0.001. Results were presented as mean ± SD. *N* = 3 per group. ANOVA was performed. *P* values were calculated relative to control samples and corrected for multiple comparisons using Dunnett's test. The figure was assembled and generated in Biorender.

We chose 4 candidate proteins for investigation namely: ARL13b, *γ*-tubulin, IFT-88 and PDGF-BB. ARL13b (ciliary membrane), *γ*-tubulin (basal body), and IFT-88 (axoneme) represent distinct regions of ciliary structure as indicated. PDGF-BB is a secreted protein by endothelial cells, was found inside cilium in our recent publication (10) and is well known to be involved in vascular stability (22). We first performed western blotting with specific antibodies to assess protein levels for each of the candidate protein in the plasma. We probed for beta-actin (*β*-actin) protein, which served as housekeeping and equal loading control for westerns. Western blot analysis and quantification showed that compared to control samples, PPH, PPH + PE and PE samples showed higher levels of ARL13b, *γ*-tubulin and IFT-88 proteins, which was statistically significant (*p* < 0.05). However, there did not appear much difference between the samples for the ciliary protein levels. Interestingly, PDGF-BB protein is the only target protein that showed lower expression levels in PPH, PPH + PE and PE samples compared to control samples. In PPH sample, the PDGF-BB protein appeared lowest (∼1-fold reduction, *p* < 0.0001) compared to controls and PPH + PE and PE samples (Figure 1).

### Quantitative PDGF-BB ELISA assay shows 3-fold reduction in PDGF-BB levels in PPH samples

**Figure 2:**
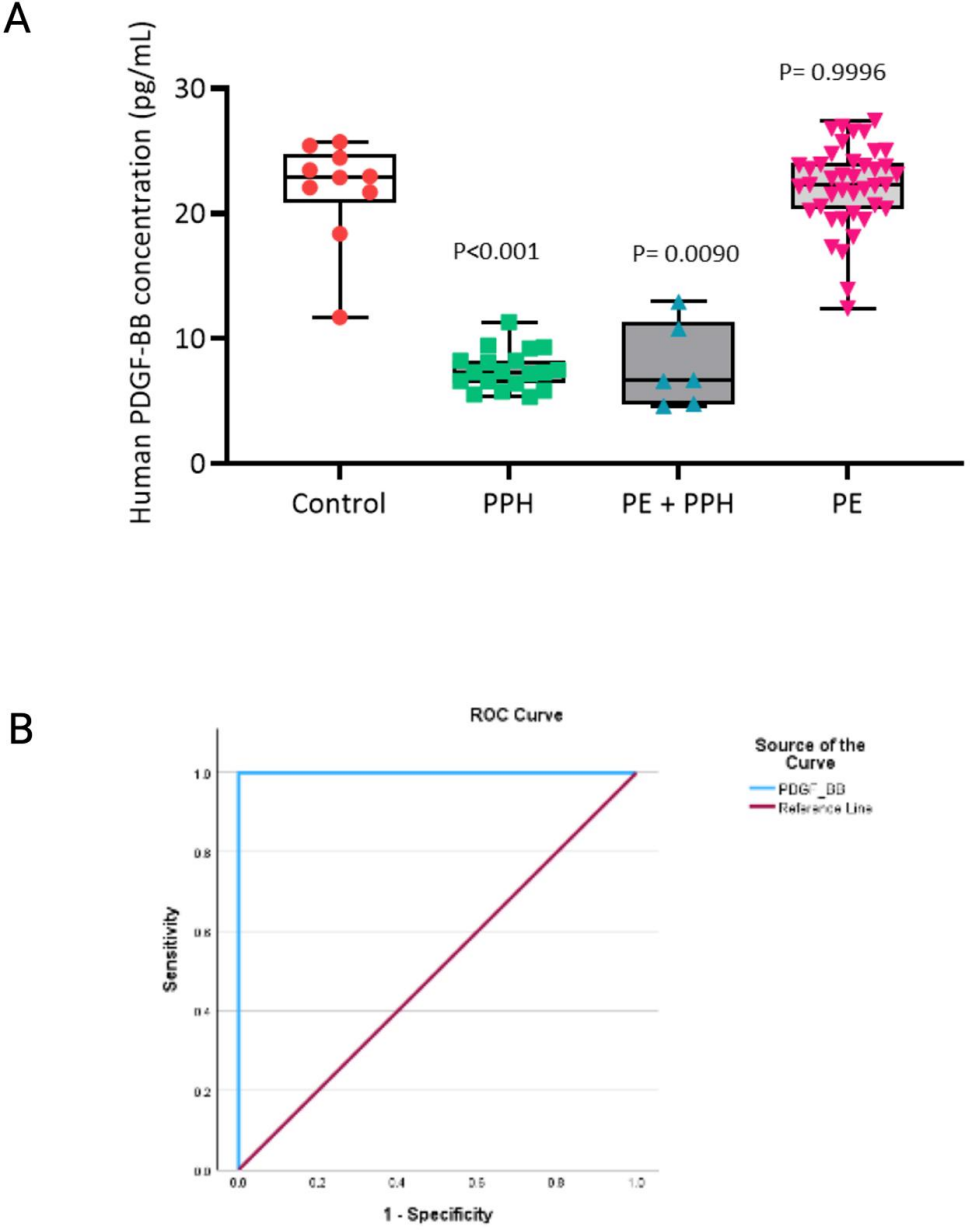
ELISA assay for PDGF-BB protein levels in human plasma samples. **(A)** ELISA analysis was performed for control (*n* = 10), PPH (*n* = 23), PPH + PE (*n* = 6), and PE (*n* = 41) human plasma samples. Representative quantification was performed by Ray Biotech's ELISA analysis tool and template provided in the manufacturer's protocol. Data are represented as mean ± SD, with statistical significance calculated against control values. Kruskal–Wallis's test was used to compare PDGF-BB among groups. Multiple comparison adjustment was done using Dunn's test. **(B)** ROC analysis was performed and Youden's index was used to identify the threshold of PDGF-BB to discriminate PPH from Control. PGDF-BB of 11.515 was the selected cutoff. The figure was assembled and generated in Biorender or SPSS.

As the Western blot is semi-quantitative in nature, we confirmed the PDGF-BB Western blot results with an ELISA RUO assay. ELISA assay results showed that PDGF-BB levels were significantly lower in PPH (median and interquartile range [7.5 (6.6, 9.3) pg/mL] and PPH + PE [6.7 (4.8, 10.8) pg/mL] compared to controls [22.9 (21.7, 24.5) pg/mL], adjusted *p* < 0.0001 and *p* = 0.0015, respectively. PE alone group (22.3 pg/mL) showed no significant difference from controls (Figure 2A). Higher PDGF-BB levels were associated with decreased risk of PPH in both unadjusted and BMI-adjusted models (adjusted OR 0.76, 95% CI 0.64–0.91; *p* = 0.0025). ROC analysis demonstrated strong discriminatory performance for PDGF-BB, with an optimal threshold of 11.5 pg/mL (Figure 2B).

## Discussion

PPH remains a major contributor to maternal morbidity and mortality, and the absence of reliable predictive biomarkers limits early risk stratification (2, 23). In this study, we identify PDGF-BB as a potential candidate biomarker associated with PPH risk, with reduced circulating levels observed in affected individuals.

### The case for PDGF-BB

PDGF-BB is a growth factor with a long history for its role in vascular stability (24). PDGF-BB participates as a secretory ligand from endothelial cells and binds to its cognate platelet-derived growth factor receptor-*β* (PDGFR*β*) on pericytes (25–27). The observed reduction in PDGF-BB levels in PPH could impair endothelial integrity, weaken vessel wall, and compromise hemostasis. This mechanistic link supports the plausibility of PDGF-BB as a biomarker for bleeding risk. In our study, the correlation association between the drop in PDGF-BB levels and PPH was observed as early as 2nd trimester. Although we do not still know whether drop in PDGF-BB levels is causal to PPH, PDGF-BB's expression pattern during pregnancy offers some insight. Cytotrophoblasts (28, 29) and endothelial cells of the endometrium (30) express PDGF-BB. Cytotrophoblast invasion if insufficient or excessive can lead to increased PPH risk, which are associated with poor spiral artery remodeling (remain narrow and muscular) or placenta becoming abnormally adherent (placenta accreta spectrum). In women with abnormal uterine bleeding, PDGF-BB levels were reduced (30) in endometrial endothelial cells suggesting a role for PDGF-BB in vascular maturation. PDGF family members (PDGF-AA, PDGF-BB, PDGFR*α*, PDGFR*β*) also show cyclic expression profile in human endometrium (30, 31).

Our study here involves protein found inside cilium. Endothelial cilia are mechanosensory organelles that transduce hemodynamic shear stress into intracellular signals governing vascular permeability, barrier integrity, and mural cell recruitment. In the context of uterine-placental vasculature, these functions are especially critical. Spiral artery remodeling, decidual vessel stabilization, and placental bed perfusion all depend on precisely regulated endothelial responses to altered flow conditions. Our studies and those of others have shown that endothelial cilia are involved in the stabilization of the vasculature (9, 19). Also, ciliary disruption associated with high shear stress (21) will cause the underlying uterine vasculature to be defective. Endothelial cells secrete PDGF-BB which has been localized inside the EC-cilium (10), and acts on PDGFR*β*-expressing pericytes to promote vessel wall coverage and stability (24, 32, 33). Endothelial cilia disruption or impaired ciliary export of PDGF-BB secondary to endothelial dysfunction may cause PDGF-BB levels to drop. These effects are potentially destabilizing to the decidual and spiral artery pericyte coverage, impairing hemostatic vascular contraction, and ultimately increasing bleeding risk at delivery. This proposed axis, cilia dysfunction - reduced PDGF-BB secretion - pericyte dropout to vascular fragility to PPH, provides a biological mechanistic framework that warrants direct experimental interrogation in future studies, including primary endometrial endothelial cell models and animal models of PPH. Taken together, PDGF-BB's expression and function in multiple processes associated with vascular stability makes it a viable causal candidate for PPH risk.

Other supportive evidence for PDGF-BB PPH risk association, include identification of lower amounts of PDGF-BB in obesity/metabolic syndrome (34). Maternal obesity is a well-established risk factor for PPH (35). Even after adjusting for BMI, the association of PDGF-BB levels to PPH remained, which makes a stronger case for PDGF-BB biomarker for PPH. The clinical implication of this finding is that integrating PDGF-BB levels into current risk stratification factors for PPH is likely to help classify patients at low, medium and high risks of PPH. At present, several factors that are currently used in predictive models for assessing PPH risk are associated with clinical indicators (36). The variables often included in predictive models include parity, antenatal hemoglobin level and bleeding, gestational age above 35, fetal weight, multiple gestations, obesity, history of previous cesarean section, and placenta previa (23). Integrating PDGF-BB levels into these predictive models will assist in better risk stratification for PPH, which would lead to pre-emptive preparation for blood transfusion, rapid access to kits (uterotonic devices, compression devices & others) for preventing PPH in the patient room and alerting emergency providers of the impending crises. These preventative measures should lead to effective management of PPH.

There are several limitations to our study. First, the retrospective nature of the analysis on limited sample sets only shows association. The sample size is modest and derived from a single center, which limits the generalizability of the finding. Further, all samples were antepartum, and none were intrapartum. Prospective evidence for PDGF-BB level drop and PPH incidence is needed to determine true predictive power. In our study, the association of higher PDGF-BB levels with lower risk for PPH remained even after adjusting for pre-pregnancy BMI. Whether this association persists with other confounding factors needs to be further explored. Additionally, the observed high diagnostic performance requires validation in prospective and independent cohorts to exclude potential overfitting or missing clinically relevant variables not available or known in our cohort. We also observed some differences in fold change in PDGF-BB between controls and PPH samples in two assays (Western vs. ELISA). This is largely attributed to the qualitative nature of western methods and the quantitative nature of the ELISA. Westerns offer high specificity while ELISA offers high sensitivity. Fourth, our plasma preparation protocol (1500 × g, 10 min) reduces but does not eliminate residual platelets, a major source of PDGF-BB. While PDGF-BB in plasma is predominantly of endothelial/non-platelet origin, unlike serum, where clotting activates platelets and substantially contributes to PDGF-BB concentrations. Thus, we cannot fully exclude a platelet contribution to measured PDGF-BB levels. Future studies should employ double-centrifugation (PPP-grade) protocols to rigorously address platelet contribution to our results. Finally, the normal levels of PDGF-BB during pregnancy and at delivery needs further exploration to understand the role of PDGF-BB in the causal pathway to PPH. Future research should focus on prospective longitudinal studies that validate PDGF-BB as a predictive biomarker, define temporal changes across gestation, and assess the clinical utility in real-time risk stratification and PPH management.

In conclusion, in a retrospective sample single site study, higher PDGF-BB levels were inversely associated with PPH risk. Additional prospective studies are underway to determine whether drop in PDGF-BB levels systemically are causal to the bleeding observed in PPH patients.

## Data Availability

The original contributions presented in the study are included in the article/Supplementary Material, further inquiries can be directed to the corresponding author.
