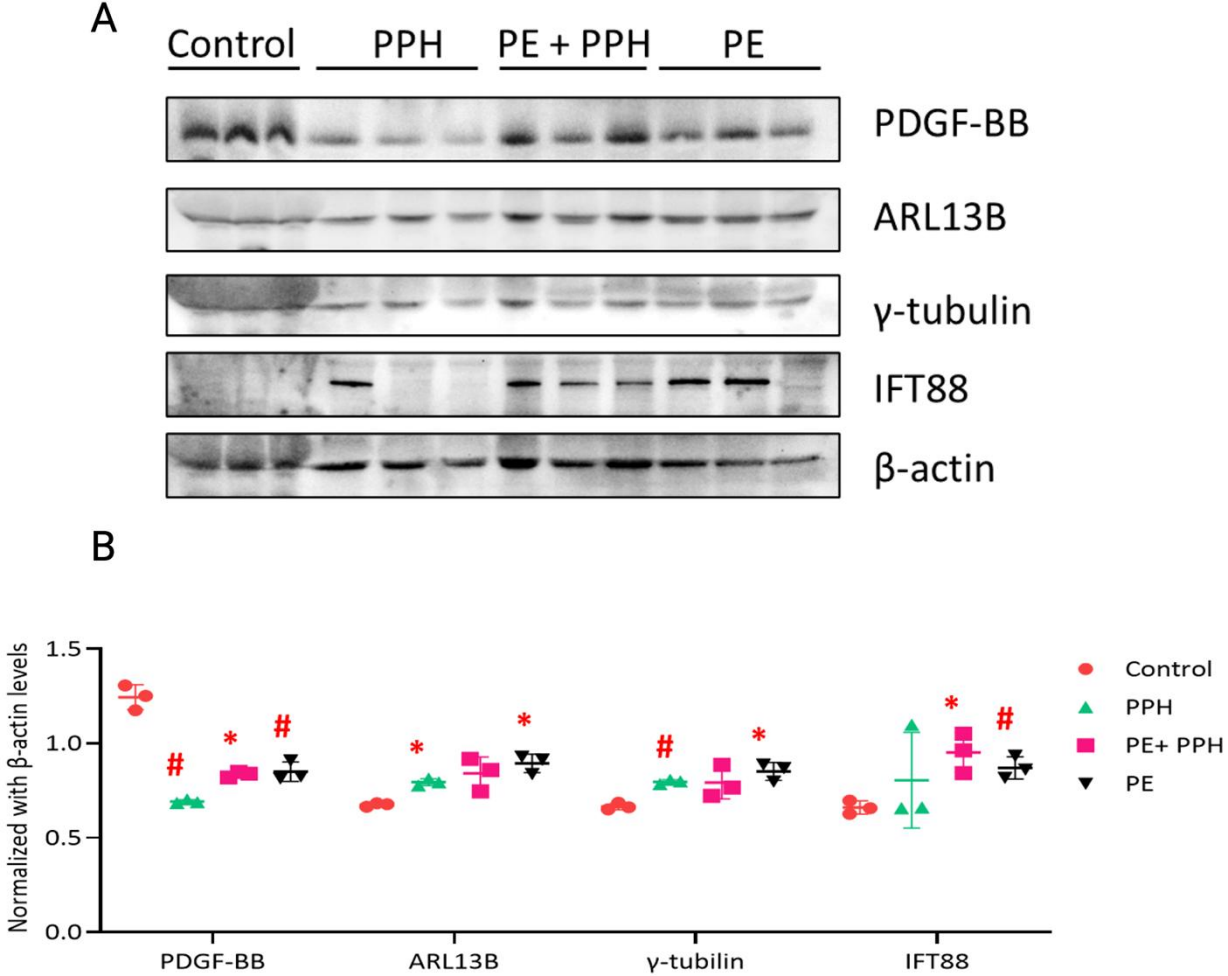# Correction: PDGF-BB as a potential biomarker for early diagnosis of postpartum hemorrhage

**DOI:** 10.3389/fgwh.2026.1913342

**Published:** 2026-07-08

**Authors:** Karthikeyan Thirugnanam, Brock E. Polnaszek, Kevin Eng, Amy Y. Pan, Ramani Ramchandran

**Affiliations:** 1 Department of Pediatrics, Division of Neonatology, Developmental Vascular Biology Program, Medical College of Wisconsin, Children’s Research Institute (CRI), Milwaukee, WI, United States; 2 Department of Obstetrics and Gynecology, Division of Maternal Fetal Medicine, Medical College of Wisconsin, Milwaukee, WI, United States; 3 CIAN, Inc., Pewaukee, WI, United States; 4 Division of Bioinformatics and Quantitative Child Health, Department of Pediatrics, Medical College of Wisconsin, CRI, Milwaukee, WI, United States

**Keywords:** biomarker, bleeding, cilia, endothelial, placenta

**Error in figure 1**

In the published article, titled, “*PDGF-BB as a potential biomarker for early diagnosis of postpartum hemorrhage*,” we noticed an error in the Figure 1, which is detailed below and needs correction.

Wrong content

There was a mistake in Figure 1 as published. The 1A panel has incorrect labels. In 1a, the panel in the published manuscript is labeled PC + PPH and PC. The correct labeling is PE + PPH and PE. The corrected Figure 1A appears below.

**Figure 1:**